# Rheumatoid arthritis and airway hyperresponsiveness: A GWAS-based mendelian randomization study

**DOI:** 10.1097/MD.0000000000048682

**Published:** 2026-05-08

**Authors:** Hengxiang Shu, Yu Wang, Ruoyi Shen, Chen Qian, Ankejiang Anniwaer, Xuwei Ling, Xu Zhu, Hao Shen, Qin Zhang

**Affiliations:** a Department of Orthopaedics, The First Affiliated Hospital of Soochow University, Soochow University, Suzhou, Jiangsu, China; b Department of Pulmonary and Critical Care Medicine, The First Affiliated Hospital of Soochow University, Suzhou, Jiangsu, China.

**Keywords:** AHDs, causal relationship, GWAS analysis, Mendelian randomization, rheumatoid arthritis

## Abstract

Previous observational studies have indicated a link between rheumatoid arthritis (RA) and airway hyperresponsiveness diseases (AHDs). However, the causality of this relationship remains uncertain. Our research aims to investigate the causal relationship between RA and AHDs. Using publicly available genome-wide association study summary statistics, we selected single nucleotide polymorphisms associated with RA (including overall RA, seropositive RA and seronegative RA), and AHDs (including asthma, bronchitis, allergic rhinitis and chronic obstructive pulmonary disease [COPD]) as instrumental variables for 2-sample Mendelian randomization (MR) analysis. The primary analytical approach was the inverse variance weighted method. Other methods, such as weighted median, weighted mode, MR-Egger regression and MR pleiotropy residual sum and outlier, were employed for quality control. We also used multiple sensitivity analysis methods to verify the robustness of our result. Genetically determined RA (odds ratio [OR] = 1.007, 95% confidence interval [CI] = 1.003–1.011, *P* < .001), seropositive rheumatoid arthritis (OR = 1.007, 95% CI = 1.002–1.011, P = .037) had a causal effect with asthma, RA (OR = 1.001, 95% CI: 1.000–1.001, P = .022), seropositive rheumatoid arthritis (OR = 1.001, 95% CI: 1.000–1.001, P < .001), or seronegative rheumatoid arthritis (OR = 1.002, 95% CI: 1.001–1.003, *P* = .001) all were associated with bronchitis risk. No obvious causal relationship was found between RA and allergic rhinitis and COPD. Sensitivity analysis did not find obvious pleiotropy or heterogeneity. The results of this study showed that genetic susceptibility to RA was causally associated with an increased risk of asthma and bronchitis but not with allergic rhinitis and COPD.

## 1. Introduction

Airway hyperresponsiveness diseases (AHDs), mainly asthma, bronchitis, allergic rhinitis, and chronic obstructive pulmonary disease (COPD), pose a significant public health challenge worldwide. These diseases cause substantial morbidity and mortality, and their prevalence is increasing globally.^[1]^ AHDs are primarily caused by a complex interplay between genetic susceptibility and environmental factors.^[2]^ Research has indicated that genetic factors play a crucial role in developing airway hyperresponsiveness and that specific genetic mutations are associated with an increased risk of developing the disease.^[3,4]^ Furthermore, environmental factors such as air pollution, allergens, and respiratory infections have also been shown to induce AHDs.^[5]^

Rheumatoid arthritis (RA) is a chronic autoimmune disorder that manifests as joint inflammation, pain, and stiffness, affecting approximately 1.71 billion people worldwide.^[6]^ Based on the presence or absence of anti-citrullinated protein antibodies and rheumatoid factors, RA can be classified into 2 subtypes: seropositive RA (POSRA) and seronegative RA (NEGRA). Several observational studies have found evidence of the association between RA and AHDs.^[7]^ However, the conclusions drawn from these studies are often inconsistent.^[8]^ More importantly, there are far fewer articles exploring potential connections between the different subtypes of RA and AHDs. Therefore, it is a key issue that needs to be urgently addressed at present to investigate RA, especially the various subtypes and their relationship with AHDs.

Mendelian randomization (MR) is an epidemiological method that uses genetic variants as instrumental variables (IVs) to assess the potential causal relationship between exposure and outcome.^[9,10]^ It can effectively avoid bias caused by confounding factors in observational studies and has been used to explore the relationship between risk factors and disease.^[11,12]^ In this study, we applied a 2-sample MR using large-scale genome-wide association studies (GWAS) to reveal the causal effect of RA on AHDs risk, including asthma, bronchitis, allergic rhinitis and COPD.

Previous studies have predominantly relied on observational designs to assess direct associations between RA and airway reactivity or structural lung damage. To our knowledge, this study represents the first 2-sample MR analysis leveraging large-scale GWAS summary statistics to dissect the causal relationships between different RA subtypes (seropositive and seronegative) and specific AHDs (asthma, bronchitis, allergic rhinitis, and COPD). By utilizing genetic variants as IVs, our approach provides unconfounded evidence regarding the differential causal effects of systemic autoimmunity on airway pathophysiology, thereby offering novel insights into the shared genetic architecture between joint and respiratory inflammation and informing targeted screening strategies for high-risk subpopulations.

## 2. Methods

### 2.1. Study design

A practical MR analysis must satisfy 3 critical assumptions: hypothesis of association: there is a strong association between single nucleotide polymorphism (SNP) and the exposure factor; hypothesis of independence: SNP is independent of confounding factors; hypothesis of exclusivity: SNP can only affect the outcome through the exposure factor.^[13]^ For the first assumption, we chose the SNP whose *P* value < 5e−8 as genetic variants. For the second assumption, we filter out the SNP related to the confounding factor through the website of PhenoScanner. For the third assumption, we delete the SNP whose *P* value > 5e−8 in the outcome. This study was based on publicly available GWAS data, and ethical approval and informed consent were obtained before the initiation of the original research.

### 2.2. GWAS dataset sources

The GWAS summary datasets for RA were obtained from the integrative epidemiology unit OpenGWAS (https://gwas.mrcieu.ac.uk/), which included 6236 cases with diagnostic information on RA. Among these cases, 4596 were POSRA, 1937 were NEGRA, and 172,834 were controls. Genetic IVs for AHDs as outcome variants encompassing asthma, bronchitis, allergic rhinitis, and COPD. GWAS were obtained from the United Kingdom Biobank (https://www.ukbiobank.ac.uk/). There were 53,598 cases and 409,335 controls for asthma; 4283 cases and 356,911 controls for allergic rhinitis; 19,320 cases and 317,839 controls for bronchitis; and 1605 cases and 461,328 controls for COPD. All cases were confirmed either by clinical laboratory testing or self-reporting. The cases and controls were derived from the European population to reduce bias in results due to the confounding effect of ethnicity. Details of all GWASs included in this study are represented in Table 1.

**Table 1:** Details of the GWASs included in the MR.

Exposure or Outcome	n	N	Population	Data source
RA	6236	147,221	European	Finngen
POSRA	4596	172,934		
NEGRA	1937	172,934		
Asthma	53,598	409,335		UK Biobank
Bronchitis	4283	356,911		
Allergic rhinitis	19,320	317,839		
COPD	1605	461,328		

COPD = chronic obstructive pulmonary disease, GWAS = genome-wide association study, MR = Mendelian randomization, n = number of cases, N = number of controls, NEGRA = seronegative rheumatoid arthritis, POSRA = seropositive rheumatoid arthritis, RA = rheumatoid arthritis, SNP = single nucleotide polymorphisms, UK = United Kingdom.

### 2.3. Quality control and identifying genetic instruments

Initially, we selected SNPs that strongly associated with RA, POSRA and NEGRA based on a *P* value threshold of less than 5e−8. Subsequently, SNPs were further subjected to clumping by restricting low linkage disequilibrium (*R*^2^ < 0.01, 10 Mb). Thirdly, we standardized the effect estimates for both exposure and outcome variants and removed any possible palindromic SNPs. Then, we used the PhenoScanner V2 database (https://doi.org/10.1093/bioinformatics/btw373) to remove SNPs associated with potential confounders.^[14]^ The potential confounders excluded from the analysis included hypertension, heart disease, autoimmune diseases, inflammation-related cells, and other lung-related conditions. The *F*-statistic for each SNP was calculated using an online tool at https://sb452.shinyapps.io/overlap, whereby a higher *F*-statistic indicates a more potent instrument.^[15]^

### 2.4. MR analyses

**Figure 1.**
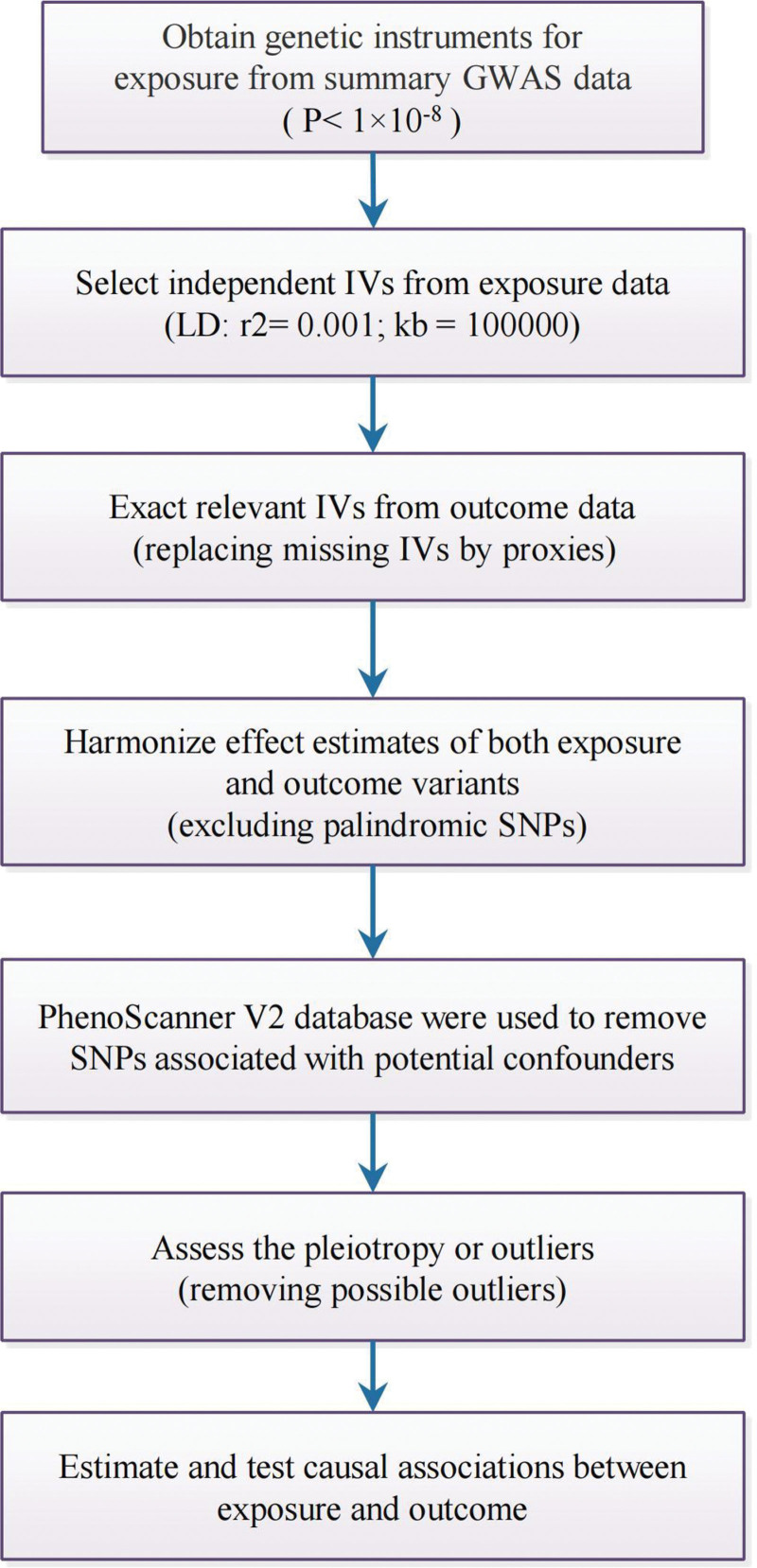
The workflow of instrumental variable analysis was used to assess the potential causal relationship between exposure and outcome. GWAS = genome-wide association study, IVs = instrumental variables, LD = linkage disequilibrium, SNPs = single nucleotide polymorphisms.

In this study, we utilized several MR analysis methods to estimate the causal effects of RA on AHDs. These methods included the Inverse Variance Weighted (IVW), MR-Egger regression, weighted median, weighted mode, and simple mode methods. The IVW method was employed as the primary analysis method. The Wald ratio method was applied when only 1 IV was available. A *P* value < .05 indicated suggestive evidence for potential association during the execution of MR analyses. We estimated the effect sizes by calculating the IVs’ odd ratio (OR) and standard error. We applied the MR-Egger regression to assess the possibility of horizontal pleiotropy, which measures the average pleiotropic effect of the IVs through the intercept ter. In addition, we used the MR-Radial and MR-pleiotropy residual sum and outlier (PRESSO) test to detect and remove any horizontal pleiotropic outliers that might bias the results.^[16]^ To evaluate the heterogeneity in the estimates, we employed the MR-Egger regression and quantified it using Cochran *Q* test.^[17]^ Furthermore, to ascertain the stability and coherence of the causal association between RA and AHDs, we carried out a leave-one-out analysis.^[18]^ The diagram outlining our approach for incorporating IVs can be found in Figure 1.

All the analyses were conducted using the “TwoSampleMR”^[19]^ and “MR-PRESSO” packages in R version 4.2.3. A *P* value below .05 was considered compelling evidence of causal relationships.

## 3. Results

### 3.1. Causal effects of RA on asthma

For the analysis of asthma, we selected 12, 7, and 4 independent SNPs with significant *P* values less than 1 × 10^-8^ as IVs for RA, POSRA, and NEGRA, respectively. These genetic variants did not have intermediate allele frequencies or palindromic structures. However, we could not include 2 RA-associated SNPs and 1 POSRA-associated SNP in the pooled analysis of asthma, as they were unavailable, and no suitable proxy SNPs were found to replace them. After excluding outliers detected in the MR-PRESSO global test, we finally used 7, 4, and 3 independent SNPs as IVs for RA, POSRA, and NEGRA, respectively, for the subsequent MR analysis.

**Figure 2.**
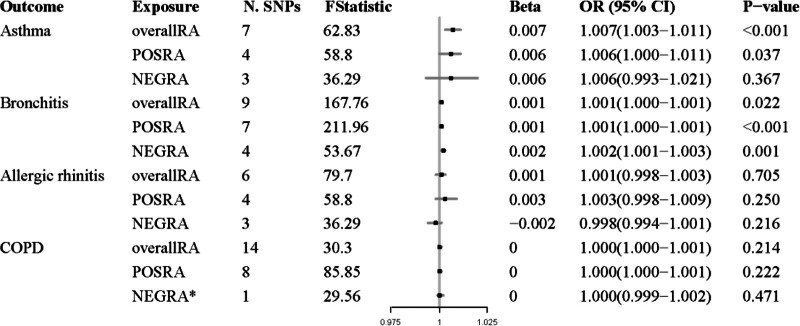
Estimated causal effects between rheumatoid arthritis and airway hyperresponsiveness diseases. CI = confidence interval, COPD = chronic obstructive pulmonary disease, NEGRA = seronegative rheumatoid arthritis, nSNP = number of single nucleotide polymorphisms, OR = odds ratio, POSRA = seropositive rheumatoid arthritis, RA = rheumatoid arthritis. * Using Wald ratio method.

**Figure 3.**
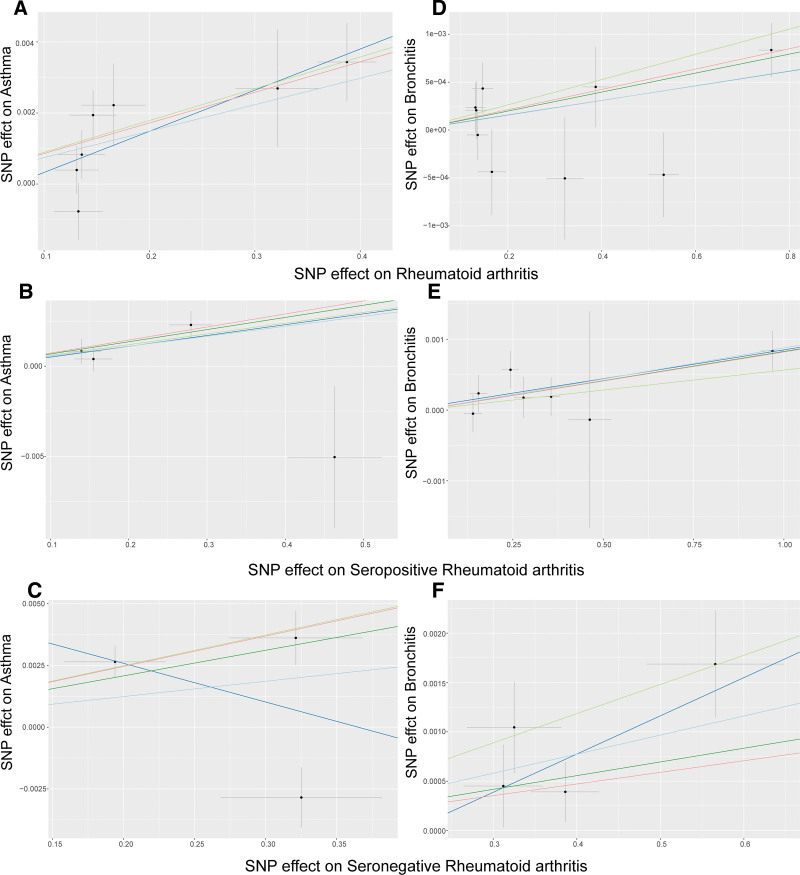
Scatter plot of the causal relationships between RA, POSRA, NEGRA and airway hyperresponsiveness diseases using different MR methods. Causal estimates for (A) RA on asthma; (B) POSRA on asthma; (C) NEGRA on RA; (D) RA on bronchitis; (E) POSRA on bronchitis; (F) NEGRA on bronchitis. MR = mendelian randomization, NEGRA = seronegative rheumatoid arthritis, POSRA = seropositive rheumatoid arthritis, RA = rheumatoid arthritis.

The results of the study showed that the IVW, weighted median, simple mode, and weighted mode methods all revealed a significant causal relationship between RA and asthma. Specifically, the IVW method yielded an OR of 1.007 with a 95% confidence interval (CI) of 1.003–1.011 and a *P* value < .001 (Fig. 2). Similar results were obtained in the weighted median method (OR = 1.009, 95% CI = 1.004–1.013, *P* < .001), simple mode method (OR = 1.009, 95% CI = 1.003–1.016, *P* = .035), and weighted mode method (OR = 1.009, 95% CI = 1.003–1.014, *P* = .018) (Table S1). Similarly, a causal relationship between POSRA and asthma was also observed (IVW, OR = 1.007, 95% CI = 1.002–1.011, *P* = .037; weighted median, OR = 1.007, 95% CI = 1.002–1.011, *P* = .003). However, no causal link was found between NEGRA and asthma in this study (IVW, OR = 1.006, 95% CI = 0.992–1.020, *P* = .367). Scatter plots of the effect sizes of SNPs for RA and its subtypes and those for arthritis are presented in Figure 3A–3C. Furthermore, the MR-Egger regression indicated that horizontal pleiotropy was unlikely to have skewed the causal relationships, as indicated by the results (RA: *P* = .404; POSRA, *P* = .964; NEGRA, *P* = .607). Cochran *Q* test showed no heterogeneity in the risk of RA, POSRA, or NEGRA and asthma (Table 2). Moreover, Table S2 shows detailed information on the final IVs used in the MR analysis of RA and asthma. The leave-one-out analysis suggested that any single SNP did not drive the causal estimates of RA and NEGRA on asthma. However, the causal relationship between POSRA and asthma is unstable due to being influenced by multiple SNPs (Fig. S1A–S1C).

**Table 2:** Sensitivity analysis of RA with airway hyperresponsiveness diseases.

Outcome	Exposure	nSNPs	MR-Egger intercept	*P* value of MR-Egger test	*P* value of Cochran *Q* statistics	*F*-Statistic
Asthma	RA	7	−8.29e−04	.404	.224	62.83
	POSRA	4	−1.01e−04	.964	.158	58.8
	NEGRA	3	5.72e−03	.607	.0	36.29
Bronchitis	RA	9	3.70e−06	.986	.265	167.76
	POSRA	7	3.52e−05	.85	.832	211.96
	NEGRA	4	−7.75e−04	.579	.318	53.67
Allergic rhinitis	RA	6	2.89e−04	.676	.388	79.7
	POSRA	4	1.75e−03	.398	.049	58.8
	NEGRA	3	3.77e−04	.912	.299	36.29
COPD	RA	14	1.82e−05	.836	.777	30.3
	POSRA	8	−4.55e−05	.703	.217	85.85
	NEGRA	1	NA	NA	NA	29.56

COPD = chronic obstructive pulmonary disease, MR = Mendelian randomization, NA = not applicable, NEGRA = seronegative rheumatoid arthritis, POSRA = seropositive rheumatoid arthritis, RA = rheumatoid arthritis, nSNP = single nucleotide polymorphism.

### 3.2. Causal effects of arthritis on bronchitis

In the MR analysis of bronchitis, we ultimately included 9, 7, and 4 independent SNPs as IVs for RA, POSRA, and NEGRA, the overall *F*-statistic for all SNPs was 167.76, 211.96, and 53.67 (Table S3), respectively. The main results of the IVW method showed a statistically significant relationship between RA (whether POSRA or NEGRA) and increased risk of bronchitis (RA: OR = 1.001, 95% CI: 1.000–1.001, *P* = .022; POSRA: OR = 1.001, 95% CI: 1.000–1.001, *P*<.001; NEGRA: OR = 1.002, 95% CI: 1.001–1.003, *P* = .001, Fig. 2). The causal estimates obtained from the other 4 MR analysis methods were consistent with IVW in terms of direction (Fig. 3D–3F and Table S1). Leave-one-out analysis indicated that the effect estimates of RA on bronchitis were influenced by 1 single SNP (rs34434863), while the causal relationship of RA on bronchitis remained robust (Fig. S1D–S1F). The MR-Egger intercept test did not show a significant deviation from zero, indicating no directional pleiotropy (Table 2). Cochran *Q* test revealed no heterogeneity in the causal estimates of RA, POSRA, or NEGRA on bronchitis (Table 2).

### 3.3. Causal effects of arthritis on allergic rhinitis

After removing outliers, 6, 3, and 4 IVs were included in the MR analysis to investigate the causal effects of RA, POSRA, and NEGRA on allergic rhinitis; the overall *F*-statistic for all SNPs was 79.7, 58.8, and 36.29 (Table S4). The results of IVW showed no significant causal relationship between RA, POSRA, or NEGRA and allergic rhinitis (RA: OR = 1.001, 95% CI: 0.998–1.003, *P* = .705; POSRA: OR = 1.003, 95% CI: 0.998–1.009, *P* = .25; NEGRA, OR = 0.998, 95% CI: 0.994–1.001, *P* = .216. Fig. 2). The results of MR-Egger, weighted median, simple mode, and weighted mode methods were consistent with those obtained from the IVW method (Fig. S2A–S2C, Table S1). There was no significant evidence of intercept, indicating the absence of directional pleiotropy (Table 2). The leave-one-out analysis demonstrated that no single SNP significantly influenced the overall outcome of RA on allergic rhinitis (Fig. S3A–S3C). Therefore, the study found no evidence of a causal relationship between RA, POSRA, or NEGRA and allergic rhinitis.

### 3.4. Causal effects of arthritis on COPD

For the analysis of COPD, we have identified 14 SNPs that serve as IVs for RA, 8 for POSRA, and 1 for NEGRA, as presented in Table S5. Employing the IVW method, we found no causal association between RA, POSRA, and COPD (RA: OR = 1.000, 95% CI: 1.000–1.001, *P* = .214; POSRA: OR = 1.000, 95% CI: 1.000–1.001, *P* = .222, Fig. 2, Fig. S2D–S2E). The heterogeneity tests conducted for RA and POSRA showed no significant evidence of heterogeneity, with P-values of 0.777 and 0.703, respectively (Table 2). The MR-Egger intercept was proximal to zero, and the *P* value was > .05, indicating the absence of significant pleiotropy. The leave-one-out analysis demonstrated that no single SNP significantly influenced the overall outcome of RA or POSRA on COPD (Fig. S3D–S3E). Correspondingly, the MR analysis by Wald ratio revealed that NEGRA negatively correlated with COPD (OR = 1.000, 95% CI: 0.999–1.002, *P* = .471 (Fig. 2). As included only a single SNP in the study on the causal effects of arthritis on COPD, sensitivity analysis was not performed.

## 4. Discussion

Our 2-sample MR analysis results provide evidence of a positive causal relationship between RA, POSRA, and asthma while also showing that RA, POSRE, or NEGRA have a causal link with bronchitis. However, there is no evidence of a causal relationship between RA and other AHDs, including allergic rhinitis and COPD. It is important to note that we have found a causal relationship between POSRA and both asthma and bronchitis. However, there is no causal relationship between NEGRA and these diseases. This result also indicates that patients with positive RA, compared to those with NEGRA, need to be more vigilant in protecting against upper airway hyperreactivity diseases.

Previous observational studies have reported an association between RA and airway-related health outcomes. For instance, a propensity-matched population study conducted in Korea revealed that patients with RA had a higher risk of developing diseases such as asthma and allergic rhinitis.^[20]^ Another study collected data from 6695 patients with RA and matched them with 26,780 individuals in the control group; the prevalence of asthma was significantly higher in the RA group, with approximately 16.4% (1095/6695) compared to 13.0% (3469/26,780) in the control group (*P* < .001). After adjusting for confounding factors, the RA group exhibited a higher asthma incidence than the control group (adjusted hazard ratio = 1.23, 95% CI = 1.15–1.32, *P* < .001).^[21]^ A cross-sectional study that utilized administrative health data included 24,625 RA patients and 25,396 individuals in the control group; the incidence rate of COPD was higher in the RA group compared to the control group (incidence rate ratio 1.58, 95% CI 1.34–1.87).^[22]^ Nevertheless, the results of these observational studies concerning the relationship between RA and AHD are only sometimes consistent. The results of a large-scale population survey indicate a significant positive correlation between RA and asthma (OR 2.32; 95% CI 1.51–3.57) and allergic rhinitis (OR 1.51; 95% CI 1.08–2.10) but no correlation with COPD (OR 0.69; 95% CI 0.45–1.06).^[8]^ Above all, these articles all support our conclusion that RA has a strong correlation with asthma and bronchitis. By leveraging GWAS data and MR techniques, we were able to further confirm the causal effect of RA on these diseases. However, there are some observational studies that do not support our findings that RA has no causal effect on COPD and allergic rhinitis. It is proposed that several explanations for these observed discrepancies. First, the positive associations reported in previous cohort studies likely reflect residual confounding by environmental factors, particularly cigarette smoking, which is strongly associated with both RA and COPD but often inadequately adjusted for in observational designs. Second, corticosteroid use in RA patients may increase susceptibility to respiratory infections and airflow limitation, thereby generating spurious associations that our MR approach effectively circumvents.

Thus, while our findings confirm the causal link between systemic autoimmunity (RA) and inflammatory airway diseases (asthma/bronchitis), they suggest that the relationships with COPD and allergic rhinitis observed in epidemiological studies may be non-causal, driven by confounding, or mediated by distinct pathophysiological pathways not captured by RA genetic risk variants.

Several potential explanations can shed light on the association between RA and AHDs. Firstly, RA’s systemic inflammation and immune dysregulation may contribute to developing AHDs^[23]^; this is attributable to the heightened activity and expression of helper T cells 17 and interleukin 17 in RA patients.^[24,25]^ These factors can instigate eosinophilic expansion, enhance airway hypersensitivity, and engender AHDs such as asthma, allergic rhinitis, and COPD.^[26–28]^ Secondly, it has been discovered that certain gene variants related to the immune system can increase genetic susceptibility to both RA and AHD. Examples of such variants include human leukocyte antigen – DR beta 1, cluster of differentiation 40 ligand and cluster of differentiation 86.^[29–31]^ Furthermore, using particular RA medications can lead to pulmonary complications, such as methotrexate.^[32]^

Nevertheless, akin to most MR studies, this study has a few limitations that warrant acknowledgments. The exposure and outcome analyzed in this study were derived from European populations, which raises questions about external validity across other ethnicities. Additionally, only a single SNP was chosen as the IVs for NEGRA and COPD, which may only explain a small part of the variation in exposure and potentially impact the statistical power of causal inference. Our study provides genetic evidence that RA, particularly POSRA, causally increases the risk of asthma and bronchitis. These findings suggest shared inflammatory pathways between RA and airway diseases, highlighting the potential for integrated therapeutic strategies targeting common immune mechanisms. Furthermore, our results underscore the importance of routine airway monitoring in RA patients, especially seropositive cases, to enable early detection and intervention.

Despite identifying causal associations between RA and certain AHDs through MR, our study has several key limitations. First, the findings are currently restricted to European populations, and their generalizability requires validation in larger, more diverse cohorts, including Asian populations. Second, the potential mediating factors or biological pathways (e.g., proteomic or metabolic mechanisms) linking RA to AHDs remain to be further elucidated. Third, further stratification by other autoantibodies, such as anti-cyclic citrullinated peptide, is needed to clarify the specific associations between RA subtypes and AHDs. Last but not least, smoking is an important risk factor for both RA and airway disease. MR studies are inherently robust to confounding by lifestyle factors due to the random assortment of alleles at conception. Although we have excluded the possibility of horizontal pleiotropy via smoking-related genetic variants using PhenoScanner, residual smoking-related pleiotropy cannot be entirely ruled out. Whether smoking acts as a mediator or effect modifier that amplifies the causal effect of RA on AHDs warrants further investigation through multivariable MR, mediation MR, or stratified MR analyses in future studies.

## 5. Conclusion

In conclusion, this MR study provides genetic evidence supporting a causal relationship between RA and asthma and bronchitis, but not allergic rhinitis and COPD. Our results highlight the importance of clinicians paying attention to respiratory symptoms in patients with RA to ensure early detection of AHDs. Further inquiry is crucial to pinpoint the precise pathogenic mechanisms by which RA heightens the likelihood of AHDs, intending to formulate targeted intervention approaches.

## Acknowledgments

The authors gratefully acknowledge support from the following funding sources: National Natural Science Foundation of China (No. 82402763).

## Author contributions

**Supervision:** Qin Zhang.

**Validation:** Hengxiang Shu, Ruoyi Shen, Chen Qian, Anniwaer Ankejiang, Xuwei Ling, Xu Zhu, Hao Shen, Qin Zhang.

**Visualization:** Yu Wang, Ruoyi Shen, Chen Qian, Anniwaer Ankejiang, Xuwei Ling, Xu Zhu, Hao Shen, Qin Zhang.

**Writing – original draft:** Hengxiang Shu, Yu Wang.

**Writing – review & editing:** Yu Wang, Qin Zhang.
















