# Supplementary material for: Rheumatoid arthritis and airway hyperresponsiveness: A GWAS-based mendelian randomization study
Source: Medicine (Baltimore). 2026 May 8;105(19):e48682. doi: 10.1097/MD.0000000000048682 (PMC13166720; doi:10.1097/MD.0000000000048682)
Supplement: Supplementary file 1 [file medi-105-e48682-s001.docx]

| **Outcome** | **Exposure** | **N.SNP** | **FStatistic** | **MR method** | **Beta** | **SE** | **OR** | **OR_lci95** | **OR_uci95** | **OR (95% CI)** | **P-value** |
| --- | --- | --- | --- | --- | --- | --- | --- | --- | --- | --- | --- |
| Asthma | overallRA | 7 | 62.83 | MR Egger | 0.012 | 0.005 | 1.012 | 1.002 | 1.022 | 1.012 (1.002-1.022) | 0.068 |
|  |  |  |  | Weighted median | 0.009 | 0.002 | 1.009 | 1.004 | 1.013 | 1.009 (1.004-1.013) | <0.001 |
|  |  |  |  | IVW | 0.007 | 0.002 | 1.007 | 1.003 | 1.011 | 1.007 (1.003-1.011) | <0.001 |
|  |  |  |  | Simple mode | 0.009 | 0.003 | 1.009 | 1.003 | 1.016 | 1.009 (1.003-1.016) | 0.035 |
|  |  |  |  | Weighted mode | 0.009 | 0.003 | 1.009 | 1.003 | 1.014 | 1.009 (1.003-1.014) | 0.019 |
|  | POSRA | 4 | 58.8 | MR Egger | 0.006 | 0.01 | 1.006 | 0.987 | 1.025 | 1.006 (0.987-1.025) | 0.602 |
|  |  |  |  | Weighted median | 0.007 | 0.002 | 1.007 | 1.002 | 1.011 | 1.007 (1.002-1.011) | 0.003 |
|  |  |  |  | IVW | 0.006 | 0.003 | 1.006 | 1.000 | 1.011 | 1.006 (1.000-1.011) | 0.037 |
|  |  |  |  | Simple mode | 0.006 | 0.003 | 1.006 | 1.000 | 1.012 | 1.006 (1.000-1.012) | 0.158 |
|  |  |  |  | Weighted mode | 0.007 | 0.002 | 1.007 | 1.002 | 1.012 | 1.007 (1.002-1.012) | 0.061 |
|  | NEGRA | 3 | 36.29 | MR Egger | -0.016 | 0.032 | 0.984 | 0.925 | 1.048 | 0.984 (0.925-1.048) | 0.709 |
|  |  |  |  | Weighted median | 0.01 | 0.003 | 1.01 | 1.004 | 1.016 | 1.01 (1.004-1.016) | 0.001 |
|  |  |  |  | IVW | 0.006 | 0.007 | 1.006 | 0.993 | 1.02 | 1.006 (0.993-1.02) | 0.367 |
|  |  |  |  | Simple mode | 0.012 | 0.003 | 1.013 | 1.006 | 1.019 | 1.013 (1.006-1.019) | 0.057 |
|  |  |  |  | Weighted mode | 0.012 | 0.003 | 1.012 | 1.006 | 1.019 | 1.012 (1.006-1.019) | 0.064 |
| Bronchitis | overallRA | 9 | 167.76 | MR Egger | 0.001 | 0.001 | 1.001 | 1.000 | 1.002 | 1.001 (1.000-1.002) | 0.218 |
|  |  |  |  | Weighted median | 0.001 | 0 | 1.001 | 1.000 | 1.002 | 1.001 (1.000-1.002) | 0.005 |
|  |  |  |  | IVW | 0.001 | 0 | 1.001 | 1.000 | 1.001 | 1.001 (1.000-1.001) | 0.022 |
|  |  |  |  | Simple mode | 0.001 | 0.001 | 1.001 | 1.000 | 1.003 | 1.001 (1.000-1.003) | 0.167 |
|  |  |  |  | Weighted mode | 0.001 | 0 | 1.001 | 1.000 | 1.002 | 1.001 (1.000-1.002) | 0.029 |
|  | POSRA | 7 | 211.96 | MR Egger | 0.001 | 0 | 1.001 | 1.000 | 1.002 | 1.001 (1.000-1.002) | 0.098 |
|  |  |  |  | Weighted median | 0.001 | 0 | 1.001 | 1.000 | 1.001 | 1.001 (1.000-1.001) | 0.002 |
|  |  |  |  | IVW | 0.001 | 0 | 1.001 | 1.000 | 1.001 | 1.001 (1.000-1.001) | <0.001 |
|  |  |  |  | Simple mode | 0.001 | 0.001 | 1.001 | 0.999 | 1.002 | 1.001 (0.999-1.002) | 0.341 |
|  |  |  |  | Weighted mode | 0.001 | 0 | 1.001 | 1.000 | 1.001 | 1.001 (1.000-1.001) | 0.020 |
|  | NEGRA | 4 | 53.67 | MR Egger | 0.004 | 0.003 | 1.004 | 0.998 | 1.01 | 1.004 (0.998-1.010) | 0.328 |
|  |  |  |  | Weighted median | 0.001 | 0.001 | 1.001 | 1.000 | 1.003 | 1.001 (1.000-1.003) | 0.042 |
|  |  |  |  | IVW | 0.002 | 0.001 | 1.002 | 1.001 | 1.003 | 1.002 (1.001-1.003) | 0.001 |
|  |  |  |  | Simple mode | 0.003 | 0.001 | 1.003 | 1.001 | 1.005 | 1.003 (1.001-1.005) | 0.072 |
|  |  |  |  | Weighted mode | 0.001 | 0.001 | 1.001 | 1.000 | 1.003 | 1.001 (1.000-1.003) | 0.226 |
| Allergic rhinitis | overallRA | 6 | 79.7 | MR Egger | -0.001 | 0.003 | 0.999 | 0.994 | 1.005 | 0.999 (0.994-1.005) | 0.856 |
|  |  |  |  | Weighted median | -0.001 | 0.002 | 0.999 | 0.996 | 1.002 | 0.999 (0.996-1.002) | 0.532 |
|  |  |  |  | IVW | 0.001 | 0.001 | 1.001 | 0.998 | 1.003 | 1.001 (0.998-1.003) | 0.705 |
|  |  |  |  | Simple mode | -0.001 | 0.003 | 0.999 | 0.994 | 1.005 | 0.999 (0.994-1.005) | 0.864 |
|  |  |  |  | Weighted mode | -0.001 | 0.002 | 0.999 | 0.996 | 1.002 | 0.999 (0.996-1.002) | 0.622 |
|  | POSRA | 4 | 58.8 | MR Egger | -0.005 | 0.008 | 0.995 | 0.979 | 1.011 | 0.995 (0.979-1.011) | 0.600 |
|  |  |  |  | Weighted median | 0.002 | 0.002 | 1.002 | 0.998 | 1.006 | 1.002 (0.998-1.006) | 0.403 |
|  |  |  |  | IVW | 0.003 | 0.003 | 1.003 | 0.998 | 1.009 | 1.003 (0.998-1.009) | 0.250 |
|  |  |  |  | Simple mode | 0.008 | 0.005 | 1.008 | 0.998 | 1.019 | 1.008 (0.998-1.019) | 0.204 |
|  |  |  |  | Weighted mode | 0 | 0.002 | 1.000 | 0.996 | 1.005 | 1.000 (0.996-1.005) | 0.940 |
|  | NEGRA | 3 | 36.29 | MR Egger | -0.004 | 0.011 | 0.996 | 0.976 | 1.017 | 0.996 (0.976-1.017) | 0.783 |
|  |  |  |  | Weighted median | -0.002 | 0.002 | 0.998 | 0.993 | 1.003 | 0.998 (0.993-1.003) | 0.391 |
|  |  |  |  | IVW | -0.002 | 0.002 | 0.998 | 0.994 | 1.001 | 0.998 (0.994-1.001) | 0.216 |
|  |  |  |  | Simple mode | -0.001 | 0.003 | 0.999 | 0.993 | 1.004 | 0.999 (0.993-1.004) | 0.733 |
|  |  |  |  | Weighted mode | -0.001 | 0.003 | 0.999 | 0.993 | 1.004 | 0.999 (0.993-1.004) | 0.688 |
| COPD | overallRA | 14 | 30.3 | MR Egger | 0 | 0 | 1.000 | 0.999 | 1.001 | 1.000 (0.999-1.001) | 0.632 |
|  |  | 14 |  | Weighted median | 0 | 0 | 1.000 | 1.000 | 1.001 | 1.000 (1.000-1.001) | 0.681 |
|  |  | 14 |  | IVW | 0 | 0 | 1.000 | 1.000 | 1.001 | 1.000 (1.000-1.001) | 0.214 |
|  |  | 14 |  | Simple mode | 0 | 0 | 1.000 | 0.999 | 1.001 | 1.000 (0.999-1.001) | 0.626 |
|  |  | 14 |  | Weighted mode | 0 | 0 | 1.000 | 0.999 | 1.001 | 1.000 (0.999-1.001) | 0.865 |
|  | POSRA | 8 | 85.85 | MR Egger | 0 | 0 | 1.000 | 1.000 | 1.001 | 1.000 (1.000-1.001) | 0.398 |
|  |  | 8 |  | Weighted median | 0 | 0 | 1.000 | 1.000 | 1.001 | 1.000 (1.000-1.001) | 0.233 |
|  |  | 8 |  | IVW | 0 | 0 | 1.000 | 1.000 | 1.001 | 1.000 (1.000-1.001) | 0.222 |
|  |  | 8 |  | Simple mode | 0 | 0.001 | 1.000 | 0.999 | 1.001 | 1.000 (0.999-1.001) | 0.537 |
|  |  | 8 |  | Weighted mode | 0 | 0 | 1.000 | 1.000 | 1.001 | 1.000 (1.000-1.001) | 0.187 |
|  | NEGRA | 1 | 29.56 | Wald ratio | 0 | 0.001 | 1.000 | 0.999 | 1.002 | 1.000 (0.999-1.002) | 0.471 |

MR, mendelian randomization; RA, rheumatoid arthritis; POSRA, seropositive rheumatoid arthritis; NEGRA, seronegative rheumatoid arthritis; N.SNPs, number of SNPs used in MR; IVW, Inverse variance weighted; OR, odds ratio; CI, confidence interval; COPD, chronic obstructive pulmonary disease.
