# Supplementary material for: Rheumatoid arthritis and airway hyperresponsiveness: A GWAS-based mendelian randomization study
Source: Medicine (Baltimore). 2026 May 8;105(19):e48682. doi: 10.1097/MD.0000000000048682 (PMC13166720; doi:10.1097/MD.0000000000048682)
Supplement: Supplementary file 2 [file medi-105-e48682-s002.docx]

| **SNPs** | **Chr** | **Position** | **EA/OA** | **EAF** | **Beta** | **SE** | **P-value** | **F-statistic** |
| --- | --- | --- | --- | --- | --- | --- | --- | --- |
| Instrumental variables of RA | | | | | | | | |
| rs114484678 | 6 | 32215057 | G/T | 0.065 | 0.322 | 0.040 | 8.81E-16 | 9.234 |
| rs11571293 | 2 | 204717713 | C/T | 0.684 | 0.131 | 0.021 | 5.7E-10 | 5.506 |
| rs17805996 | 8 | 129515441 | C/T | 0.122 | 0.166 | 0.030 | 4.77E-08 | 4.259 |
| rs3129294 | 6 | 33084671 | T/G | 0.719 | 0.147 | 0.022 | 2.87E-11 | 6.335 |
| rs6679677 | 1 | 114303808 | A/C | 0.148 | 0.387 | 0.028 | 1.27E-43 | 27.332 |
| rs7574865 | 2 | 191964633 | A/G | 0.232 | 0.133 | 0.023 | 1.04E-08 | 4.667 |
| rs7731626 | 5 | 55444683 | T/C | 0.722 | 0.136 | 0.022 | 5.51E-10 | 5.493 |
| Instrumental variables of positve RA | | | | | | | | |
| rs11571293 | 2 | 204717713 | G/T | 0.589 | 0.155 | 0.024 | 1.61E-10 | 10.256 |
| rs116818505 | 6 | 33051900 | T/G | 0.719 | 0.280 | 0.027 | 2E-24 | 26.013 |
| rs142999768 | 6 | 26625991 | T/C | 0.008 | 0.463 | 0.060 | 1.49E-14 | 14.781 |
| rs7731626 | 5 | 55444683 | G/A | 0.625 | 0.140 | 0.025 | 2.46E-08 | 7.744 |
| Instrumental variables of negative RA | | | | | | | | |
| rs2476601 | 1 | 114377568 | A/G | 0.147 | 0.321 | 0.047 | 9.96E-12 | 15.436 |
| rs9261599 | 6 | 30221058 | T/G | 0.455 | 0.194 | 0.036 | 5.25E-08 | 9.853 |
| rs9296004 | 6 | 31933977 | C/A | 0.097 | 0.325 | 0.057 | 9.39E-09 | 10.997 |

SNP, single nucleotide polymorphism; Chr, chromosome; EA/OA, effect allele/other allele; EAF, effect allele frequency; SE, standard error of beta; RA, rheumatoid arthritis; POSRA, POSRA, seropositive rheumatoid arthritis.
