# Supplementary figures and images for: Rheumatoid arthritis and airway hyperresponsiveness: A GWAS-based mendelian randomization study
Source: Medicine (Baltimore). 2026 May 8;105(19):e48682. doi: 10.1097/MD.0000000000048682 (PMC13166720; doi:10.1097/MD.0000000000048682)

**
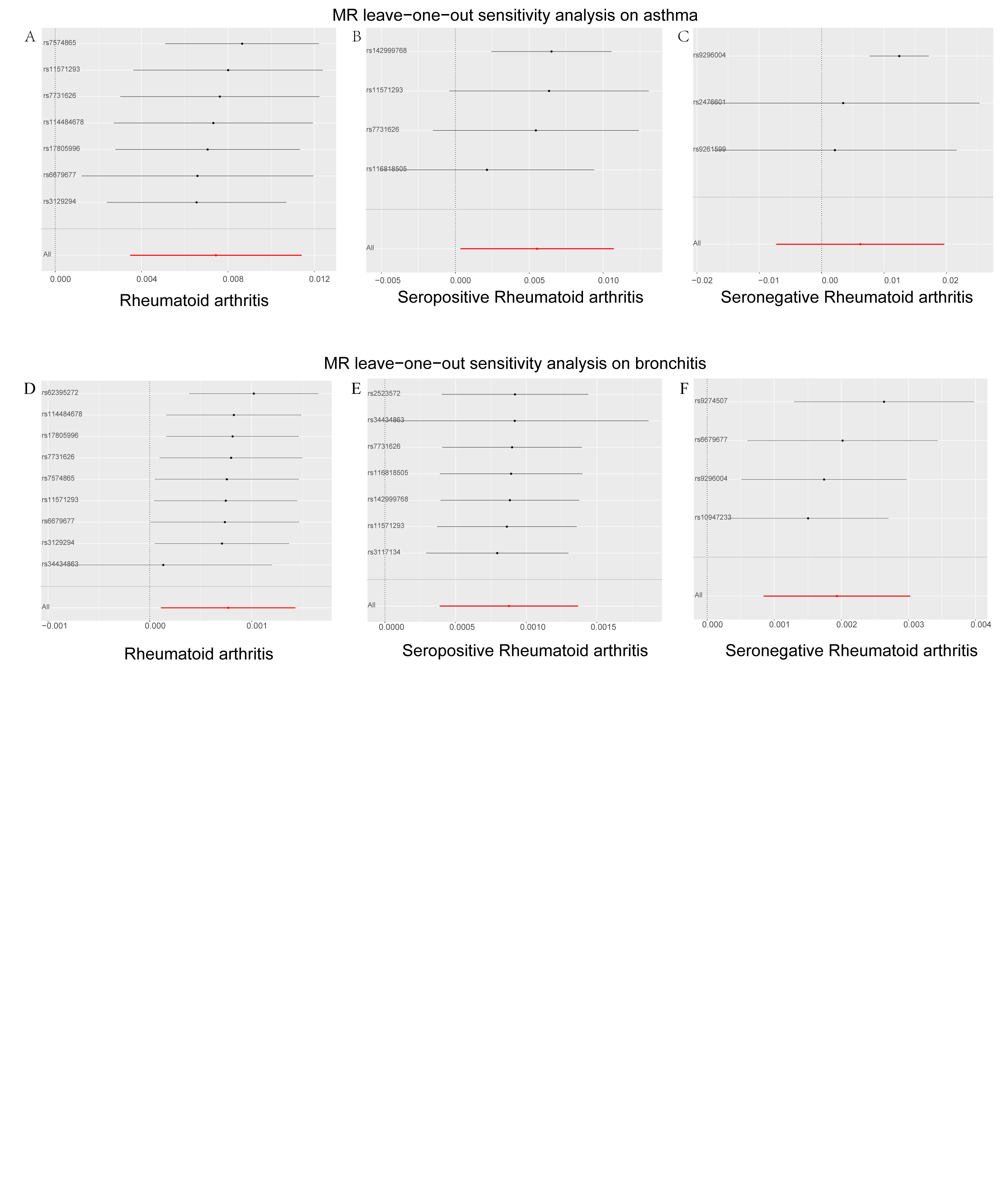
**

Supplement: Supplementary file 3 [file medi-105-e48682-s003.docx]

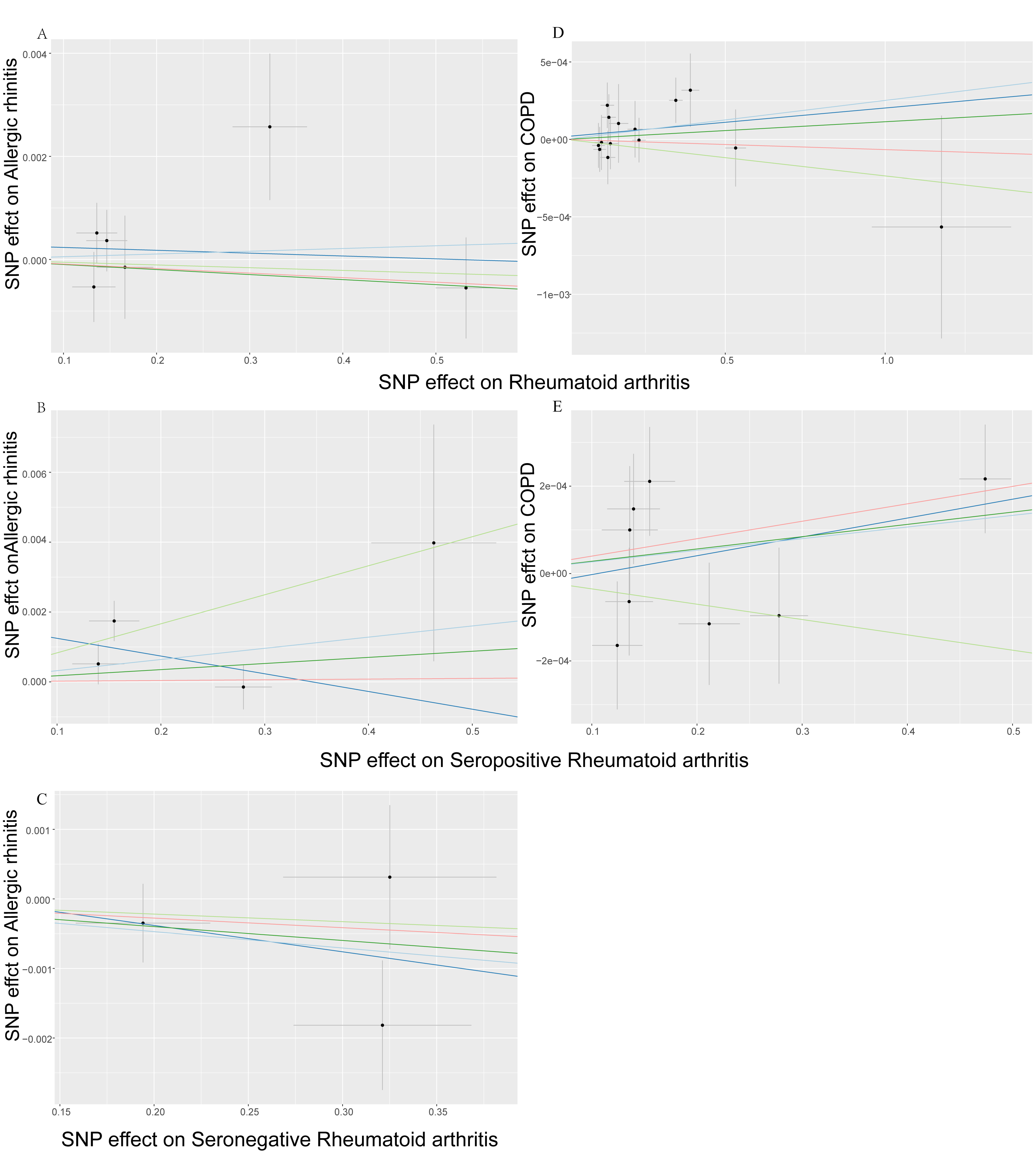

Supplement: Supplementary file 6 [file medi-105-e48682-s006.docx]

**
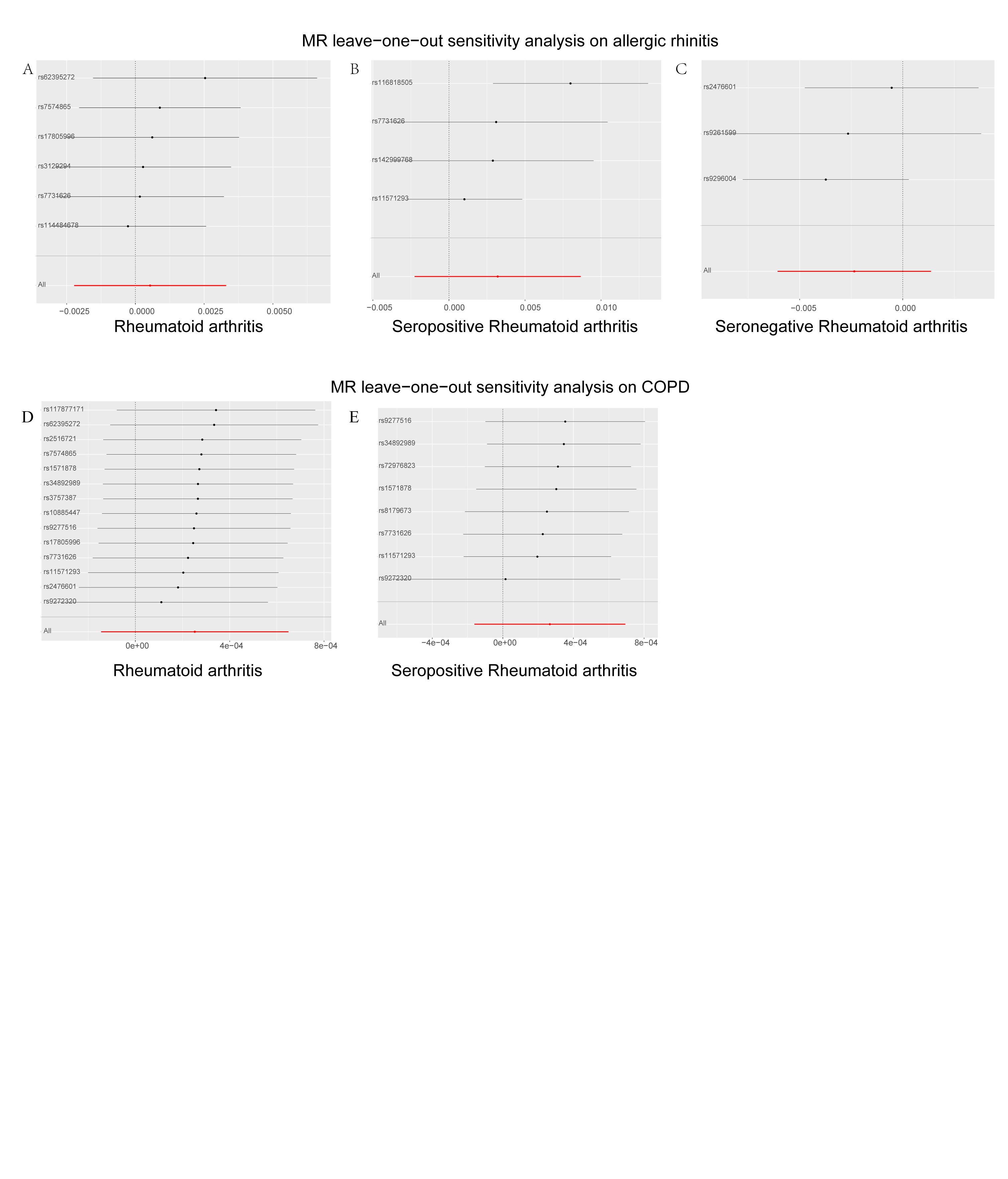
**

Supplement: Supplementary file 7 [file medi-105-e48682-s007.docx]
