# Supplementary material for: Rheumatoid arthritis and airway hyperresponsiveness: A GWAS-based mendelian randomization study
Source: Medicine (Baltimore). 2026 May 8;105(19):e48682. doi: 10.1097/MD.0000000000048682 (PMC13166720; doi:10.1097/MD.0000000000048682)
Supplement: Supplementary file 4 [file medi-105-e48682-s004.docx]

| **SNPs** | **Chr** | **Position** | **EA/OA** | **EAF** | **Beta** | **SE** | **P-value** | **F-statistic** |
| --- | --- | --- | --- | --- | --- | --- | --- | --- |
| Instrumental variables of RA | | | | | | | | |
| rs114484678 | 6 | 32215057 | C/T | 0.065 | 0.322 | 0.040 | 8.808E-16 | 7.182 |
| rs11571293 | 2 | 204717713 | G/T | 0.684 | 0.131 | 0.021 | 5.699E-10 | 4.283 |
| rs17805996 | 8 | 129515441 | C/T | 0.122 | 0.166 | 0.030 | 4.767E-08 | 3.313 |
| rs3129294 | 6 | 33084671 | A/C | 0.719 | 0.147 | 0.022 | 2.871E-11 | 4.927 |
| rs34434863 | 6 | 32559673 | G/T | 0.341 | 0.762 | 0.027 | 4.7E-176 | 89.084 |
| rs62395272 | 6 | 31394424 | T/C | 0.109 | 0.532 | 0.033 | 2.19E-60 | 29.804 |
| rs6679677 | 1 | 114303808 | A/C | 0.148 | 0.387 | 0.028 | 1.274E-43 | 21.258 |
| rs7574865 | 2 | 191964633 | T/G | 0.232 | 0.133 | 0.023 | 1.035E-08 | 3.629 |
| rs7731626 | 5 | 55444683 | G/A | 0.722 | 0.136 | 0.022 | 5.511E-10 | 4.272 |
| Instrumental variables of POSRA | | | | | | | | |
| rs11571293 | 2 | 204717713 | G/T | 0.684 | 0.155 | 0.024 | 1.61E-10 | 5.860 |
| rs116818505 | 6 | 33051900 | T/G | 0.745 | 0.280 | 0.027 | 2.001E-24 | 14.864 |
| rs142999768 | 6 | 26625991 | T/C | 0.038 | 0.463 | 0.060 | 1.495E-14 | 8.446 |
| rs2523572 | 6 | 31329494 | T/C | 0.601 | 0.356 | 0.026 | 6.253E-44 | 27.610 |
| rs3117134 | 6 | 32313550 | T/C | 0.486 | 0.244 | 0.023 | 1.16E-25 | 15.737 |
| rs34434863 | 6 | 32559673 | G/T | 0.340 | 0.968 | 0.032 | 1E-200 | 135.013 |
| rs7731626 | 5 | 55444683 | G/A | 0.720 | 0.140 | 0.025 | 2.462E-08 | 4.425 |
| Instrumental variables of NEGRA | | | | | | | | |
| rs10947233 | 6 | 32124424 | T/G | 0.047 | 0.566 | 0.082 | 5.263E-12 | 11.902296 |
| rs6679677 | 1 | 114303808 | A/C | 0.146 | 0.312 | 0.0473 | 4.321E-11 | 10.884232 |
| rs9274507 | 6 | 32634152 | A/G | 0.738 | 0.386 | 0.0406 | 1.969E-21 | 22.632363 |
| rs9296004 | 6 | 31933977 | C/A | 0.097 | 0.325 | 0.0566 | 9.394E-09 | 8.2477147 |

SNP, single nucleotide polymorphism; Chr, chromosome; EA/OA, effect allele/other allele; EAF, effect allele frequency; SE, standard error of beta; RA, rheumatoid arthritis; POSRA, POSRA, seropositive rheumatoid arthritis; NEGRA, seronegative rheumatoid arthritis.
