# Supplementary material for: Rheumatoid arthritis and airway hyperresponsiveness: A GWAS-based mendelian randomization study
Source: Medicine (Baltimore). 2026 May 8;105(19):e48682. doi: 10.1097/MD.0000000000048682 (PMC13166720; doi:10.1097/MD.0000000000048682)
Supplement: Supplementary file 8 [file medi-105-e48682-s008.docx]

| **SNPs** | **Chr** | **Position** | **EA/OA** | **EAF** | **Beta** | **SE** | **P-value** | **F-statistic** |
| --- | --- | --- | --- | --- | --- | --- | --- | --- |
| Instrumental variables of RA | | | | | | | |  |
| rs10885447 | 10 | 115166770 | G/A | 0.733 | 0.113 | 0.022 | 3.22E-07 | 1.861 |
| rs11571293 | 2 | 204717713 | G/T | 0.684 | 0.131 | 0.021 | 5.70E-10 | 2.753 |
| rs117877171 | 19 | 49427763 | C/T | 0.003 | 1.176 | 0.218 | 6.55E-08 | 2.084 |
| rs1571878 | 6 | 167540842 | C/T | 0.448 | 0.107 | 0.020 | 5.42E-08 | 2.111 |
| rs17805996 | 8 | 129515441 | C/T | 0.122 | 0.166 | 0.030 | 4.77E-08 | 2.130 |
| rs2476601 | 1 | 114377568 | A/G | 0.149 | 0.391 | 0.028 | 1.03E-44 | 14.107 |
| rs2516721 | 6 | 30222566 | C/T | 0.489 | 0.230 | 0.021 | 7.13E-28 | 8.567 |
| rs34892989 | 6 | 33799390 | G/A | 0.793 | 0.141 | 0.025 | 2.19E-08 | 2.241 |
| rs3757387 | 7 | 128576086 | C/T | 0.424 | 0.103 | 0.020 | 1.74E-07 | 1.940 |
| rs62395272 | 6 | 31394424 | T/C | 0.109 | 0.532 | 0.033 | 2.19E-60 | 19.159 |
| rs7574865 | 2 | 191964633 | T/G | 0.232 | 0.133 | 0.023 | 1.04E-08 | 2.333 |
| rs7731626 | 5 | 55444683 | G/A | 0.722 | 0.136 | 0.022 | 5.51E-10 | 2.746 |
| rs9272320 | 6 | 32605257 | A/G | 0.430 | 0.345 | 0.021 | 1.66E-59 | 18.937 |
| rs9277516 | 6 | 33054325 | T/C | 0.745 | 0.218 | 0.024 | 3.47E-20 | 6.072 |
| Instrumental variables of POSRA | | | | | | | |  |
| rs11571293 | 2 | 204717713 | G/T | 0.684 | 0.155 | 0.024 | 1.61E-10 | 5.128 |
| rs1571878 | 6 | 167540842 | C/T | 0.447 | 0.136 | 0.023 | 2.24E-09 | 4.454 |
| rs34892989 | 6 | 33799390 | G/A | 0.793 | 0.212 | 0.029 | 4.58E-13 | 6.564 |
| rs72976823 | 6 | 138019512 | G/A | 0.671 | 0.124 | 0.024 | 1.93E-07 | 3.376 |
| rs7731626 | 5 | 55444683 | G/A | 0.720 | 0.140 | 0.025 | 2.46E-08 | 3.872 |
| rs8179673 | 2 | 191969341 | C/T | 0.232 | 0.136 | 0.027 | 3.20E-07 | 3.272 |
| rs9272320 | 6 | 32605257 | A/G | 0.429 | 0.474 | 0.025 | 6.24E-83 | 46.407 |
| rs9277516 | 6 | 33054325 | T/C | 0.745 | 0.278 | 0.028 | 4.16E-24 | 12.774 |
| Instrumental variables of NEGRA | | | | | | | | |
| rs9261599 | 6 | 30221058 | T/G | 0.455 | 0.1941 | 0.036 | 5.25303E-08 | 29.561 |

SNP, single nucleotide polymorphism; Chr, chromosome; EA/OA, effect allele/other allele; EAF, effect allele frequency; SE, standard error of beta; RA, rheumatoid arthritis; POSRA, POSRA, seropositive rheumatoid arthritis; NEGRA, seronegative rheumatoid arthritis.
